# Effects of Tai Chi on fatigue and resting-state brain activity in chronic fatigue syndrome: a randomized controlled fMRI study

**DOI:** 10.3389/fnins.2026.1910307

**Published:** 2026-09-09

**Authors:** Bin Wang, Xiaodong Zhang

**Affiliations:** 1 College of Wushu, Shanghai University of Sport, Shanghai, China; 2 Department of Physical Education, Shanghai University of Traditional Chinese Medicine, Shanghai, China; 3 Department of Massage, Yueyang Hospital of Integrated Chinese and Western Medicine, Shanghai, China

**Keywords:** amplitude of low-frequency fluctuations, chronic fatigue syndrome, fatigue, randomized controlled trial, resting-state functional magnetic resonance imaging, Tai Chi

## Abstract

**Background:**

Chronic fatigue syndrome (CFS) is a complex disorder characterized by persistent fatigue, sleep disturbance, and cognitive impairment. It substantially impairs both physical and mental health. Tai Chi, a traditional Chinese mind–body exercise, may improve symptoms in patients with CFS. However, its neural mechanisms remain unclear. This study investigated the effects of Tai Chi on spontaneous brain activity in patients with CFS using resting-state functional magnetic resonance imaging (rs-fMRI) and amplitude of low-frequency fluctuations (ALFF).

**Methods:**

Sixty patients with CFS were randomly assigned in a 1:1 ratio to either a Tai Chi group or a health education group; 57 participants completed both fMRI scans and fatigue assessments and were included in the complete-case analysis. Both groups underwent a 12-week intervention consisting of three 60-min sessions per week. Adherence was monitored using weekly logs. Fatigue severity was assessed before and after the intervention using the Multidimensional Fatigue Inventory (MFI-20). Resting-state fMRI data were acquired at both time points. Preprocessing included motion correction, normalization to Montreal Neurological Institute space, and spatial smoothing. ALFF was calculated to quantify regional spontaneous neural activity. Correlation analyses were performed to assess associations between changes in ALFF and MFI-20 scores. Harms were monitored throughout the intervention period.

**Results:**

Fifty-seven participants were included in the complete-case analysis (Tai Chi, *n* = 29; health education, *n* = 28). Compared with health education, Tai Chi produced greater reductions in MFI-20 total score from baseline to week 12 (between-group change difference, −9.50 points; 95% CI, −12.31 to −6.70; Cohen's *d* = −1.83; *P* < 0.001). Greater improvements were also observed in overall fatigue, physical fatigue, mental fatigue, activity reduction, and interest reduction, with between-group differences ranging from −1.26 to −2.44 points. In imaging analyses, Tai Chi was associated with increased ALFF in prefrontal, hippocampal/striatal, and supplementary motor regions and decreased ALFF in occipital and temporal regions, using voxel-level *P* < 0.001 and cluster-level family-wise error correction. Exploratory brain–behavior analyses showed that increases in ALFF in the left medial superior frontal gyrus and left dorsolateral superior frontal gyrus were associated with greater reductions in MFI-20 scores, whereas decreases in ALFF in the left inferior occipital gyrus were associated with fatigue improvement. No falls, injuries, severe post-exertional malaise, or intervention-related medical events were reported through weekly participant logs or supervised-session monitoring.

**Conclusion:**

A 12-week Tai Chi intervention was associated with greater fatigue improvement than health education and with region-specific changes in resting-state spontaneous brain activity in patients with CFS. Tai Chi was associated with reduced fatigue and altered spontaneous brain activity in prefrontal, hippocampal, motor, and sensory-related regions in patients with CFS. These findings suggest that Tai Chi may modulate fatigue-related brain function, which may contribute to improvements in fatigue severity. Future studies should replicate and extend existing network-level findings using prespecified, adequately powered connectivity and mediation analyses with active exercise comparators and longer-term follow-up.

**Chinese clinical trial registration:**

https://www.chictr.org.cn/showproj.html?proj=223860, ChiCTR2400082268; March 26, 2024.

## 1 Background

Chronic fatigue syndrome (CFS) is a multisystem disorder characterized by persistent, disabling fatigue, post-exertional malaise, unrefreshing sleep, and cognitive impairment, typically lasting for several months, with incompletely understood underlying mechanisms (Sandler and Lloyd, 2020). Epidemiological studies estimate that 17–24 million people worldwide are affected (Lim et al., 2020). The annual cost of CFS treatment is estimated at USD 18–24 billion, representing a substantial burden on society and healthcare systems (Cochrane et al., 2021).

Current therapeutic strategies for CFS are largely symptomatic and include immunomodulation, antiviral therapy, nutritional support, psychological interventions, and cognitive behavioral therapy (Rollnik, 2017; Blitshteyn and Chopra, 2018; Rowe et al., 2001). However, these approaches have limited efficacy, may be costly, and can cause adverse effects (Rowe et al., 2001). The Centers for Disease Control and Prevention (CDC) has noted that cognitive behavioral therapy and graded exercise therapy have limited effectiveness (Bateman et al., 2021). The National Institute for Health and Care Excellence (NICE) does not recommend routine pharmacological treatment (Royal College of Physicians, 2021). These limitations highlight the need for safe, effective, and sustainable interventions.

Complementary and alternative medicine (CAM) has been increasingly used in conditions with unclear etiologies (Alraek et al., 2011). Previous studies have shown that traditional Japanese herbal medicine (Wang et al., 2004), acupuncture (Schweiger et al., 2020), and mind–body exercises (Kong et al., 2023; Khanpour Ardestani et al., 2021) hold promise in alleviating fatigue, depression, and sleep disturbances. Mind–body exercises integrate posture, breathing, and mental focus; they are low-intensity and adaptable and may improve energy metabolism while reducing the risk of post-exertional malaise (Xie et al., 2023, 2022a; Chan et al., 2019). Randomized trials have demonstrated benefits of Qigong and yoga for CFS (Oka et al., 2014, 2019). However, high-quality randomized controlled trials examining Tai Chi for CFS remain scarce internationally (Markwart et al., 2024), leaving the neural mechanisms underlying its effects largely unexplored.

From a mechanistic perspective, neuroendocrine imbalance, immune dysfunction, and central nervous system abnormalities are considered key contributors to CFS. Although the exact mechanisms are not fully understood, CFS is often regarded as a disorder associated with brain network dysfunction (Washington et al., 2020; VanElzakker et al., 2019). Core symptoms, including severe fatigue, cognitive impairment, and poor sleep quality, suggest that disruption of normal brain function plays a central role in the disease process (Shan et al., 2020; Arnett et al., 2011).

Resting-state functional magnetic resonance imaging (rs-fMRI), which measures spontaneous neural activity through blood oxygen level-dependent signals, is a non-invasive method with high spatial resolution and is widely used to investigate network dysfunction in CFS (Goense et al., 2016; Lee et al., 2013). Previous fMRI studies have reported alterations in the prefrontal cortex, parietal cortex, limbic system, and related networks involved in memory, emotional processing, and cognitive control (Gay et al., 2016; Puri et al., 2012; Caseras et al., 2008).

Tai Chi is a traditional Chinese mind–body exercise that is safe, accessible, and does not require special equipment or facilities. It is a gentle, comprehensive form of exercise and may be particularly suitable for individuals with fatigue, low physical activity, or sedentary lifestyles (Hartley et al., 2014). Tai Chi has been shown to be more effective than conventional aerobic exercise in improving symptoms of fibromyalgia (FM) (Wang et al., 2010, 2018). FM shares many clinical features with CFS and may involve similar pathophysiological mechanisms (Hulens et al., 2018). Recent studies have highlighted the neuroplastic effects of Tai Chi on brain structure and function, including increased gray matter volume, improved functional connectivity, and altered resting-state network characteristics associated with cognitive flexibility. Compared with general aerobic exercise, Tai Chi may exert greater effects on neuroplasticity and overall brain network function (Cui et al., 2019; Wei et al., 2017; Cui et al., 2021). Therefore, Tai Chi may benefit patients with CFS by modulating brain function. However, its specific neural mechanisms remain unclear.

In this study, Tai Chi was used as the intervention. rs-fMRI and amplitude of low-frequency fluctuations (ALFF) analysis were used to investigate changes in spontaneous brain activity and fatigue symptoms in patients with CFS. The aim was to provide neuroimaging evidence regarding the potential neural mechanisms of Tai Chi in CFS. Previous neuroimaging studies have already demonstrated that mind–body exercise may influence both regional spontaneous activity and large-scale functional connectivity in patients with CFS. A 12-week Prolong Life With Nine Turn Method Qigong study reported changes in ALFF and seed-based functional connectivity involving occipital, superior frontal, and cingulate regions (Xie et al., 2022b). In addition, two 1-month Tai Chi studies reported changes in large-scale brain connectivity, including altered effective connectivity between the sensorimotor and default mode networks and strengthened functional connectivity involving the default mode and left frontoparietal networks (Li et al., 2022; Wu et al., 2022). However, these Tai Chi studies used longitudinal CFS–healthy control designs in which both groups received Tai Chi and did not include a randomized non-exercise comparator. Evidence therefore remains limited regarding whether a 12-week standardized 24-form Tai Chi intervention produces fatigue-related changes in regional spontaneous neural activity in a randomized parallel-group design. The present study consequently focused on ALFF as a regional measure of spontaneous brain activity and examined whether ALFF changes were associated with changes in multidimensional fatigue symptoms.

The specific objective was to evaluate the benefits and harms of a 12-week Tai Chi intervention compared with health education on fatigue severity and resting-state spontaneous brain activity in patients with CFS. The study protocol has been published in JMIR Research Protocols (Wang et al., 2025).

## 2 Materials and methods

### 2.1 Subjects

Patients with chronic fatigue syndrome (CFS) were recruited from Shanghai University of Traditional Chinese Medicine and its affiliated Yueyang Hospital of Integrated Traditional Chinese and Western Medicine. Recruitment was conducted through notices in outpatient clinics, WeChat public accounts, and posters placed both within and outside the university. All potential participants were evaluated at Yueyang Hospital by qualified physicians according to the diagnostic criteria. Diagnosis was confirmed by one qualified physician using a standardized eligibility checklist based on the NICE 2021 criteria. Relevant laboratory tests and medical history were reviewed to exclude alternative causes of fatigue. Disagreements regarding eligibility were resolved by discussion with a senior clinician. Eligible patients were enrolled in the study. Before enrollment, the study purpose, potential benefits, and potential risks were explained to the participants. All participants provided written informed consent and were informed of their right to withdraw from the study at any time without penalty.

The clinical diagnosis of CFS was based on the 2021 diagnostic criteria of the National Institute for Health and Care Excellence (NICE) (Royal College of Physicians, 2021). The diagnosis required the presence of the following four symptoms: (1) persistent fatigue that worsens after activity and is not substantially relieved by rest; (2) cognitive dysfunction (referred to as “brain fog”), manifested as slowed responses, impaired short-term memory, difficulty concentrating, or difficulty multitasking; (3) post-exertional malaise, which typically occurs hours to days after activity, is disproportionate to the level of exertion, and is associated with prolonged recovery lasting from hours to weeks; (4) unrefreshing sleep or sleep disturbance. These symptoms had to persist for at least 3 months, and other medical causes of fatigue had to be excluded.

Inclusion criteria were as follows:

1. meeting the NICE 2021 diagnostic criteria for CFS;
2. aged 18–60 years, any sex, and right-handedness;
3. normal blood and urine test results and normal liver and kidney function;
4. no related treatment within the previous month;
5. voluntary participation with written informed consent.

Exclusion criteria were as follows:

1. failure to meet the diagnostic or inclusion criteria;
2. severe cardiovascular, cerebrovascular, or psychiatric disorders;
3. contraindications to MRI (e.g., metal implants or claustrophobia);
4. fatigue relieved by rest and not associated with functional impairment;
5. serious medical comorbidities;
6. pregnancy or breastfeeding;
7. refusal to participate.

#### 2.1.1 Patient and public involvement

Patients or members of the public were not formally involved in the design, conduct, analysis, or reporting of this trial. Before enrollment, potential participants were informed about the study purpose, potential benefits, potential risks, and their right to withdraw at any time without penalty.

### 2.2 Study design

This study used a randomized, two-arm, parallel-group controlled trial design with a 1:1 allocation ratio and an exploratory superiority framework. Eligible participants were randomly assigned to either the Tai Chi group or the health education group. The Tai Chi intervention was conducted at Shanghai University of Traditional Chinese Medicine and was led by an instructor with more than 5 years of experience. The intervention included three sessions per week: two in-person sessions and one supervised online session. The health education group received CFS-related education through a WeChat group, with three sessions per week focused on disease-related knowledge. The intervention period was 12 weeks. Participants completed weekly adherence logs.

The study was conducted in accordance with the Declaration of Helsinki (2013 revision) and relevant international ethical guidelines. Ethical approval was obtained from the Shanghai Clinical Research Ethics Committee (Approval No. SECCR2024-22-01), and the trial was registered with the Chinese Clinical Trial Registry (ChiCTR2400082268; March 26, 2024). The trial was prospectively registered with the Chinese Clinical Trial Registry on March 26, 2024, before the first participant was enrolled in April 2024.

The published protocol can be accessed in JMIR Research Protocols (Wang et al., 2025) and the trial registry entry can be accessed through the Chinese Clinical Trial Registry using the identifier ChiCTR2400082268. The present manuscript reports the fatigue and ALFF outcomes related to the neuroimaging analysis. Compared with the published protocol, functional connectivity analyses, cognitive outcomes, sleep outcomes, and longer-term follow-up assessments are not reported in this manuscript and should be reported separately or explicitly identified as outside the scope of the present analysis. The present analysis used complete-case analysis of participants with both baseline and post-intervention MFI-20 and usable fMRI data; participants with excessive head motion were excluded from imaging analyses according to the prespecified quality-control criterion.

### 2.3 Randomization and allocation concealment

After providing written informed consent, participants were randomly assigned in a 1:1 ratio to the Tai Chi group or the health education group. The allocation sequence was implemented using sequentially numbered, opaque, sealed envelopes prepared by an independent researcher who was not involved in recruitment, intervention delivery, outcome assessment, or data analysis. The envelopes were opened only after baseline assessment and confirmation of eligibility, thereby maintaining allocation concealment until assignment. Because of the nature of the intervention, participant blinding was not feasible; however, MRI preprocessing and statistical analyses were performed using coded identifiers. The personnel who enrolled participants and those who assigned participants to interventions did not have access to the random allocation sequence before assignment. Outcome assessors and data analysts were blinded by using coded participant identifiers.

### 2.4 Sample size calculation

The sample size was calculated using G^*^Power 3.1 for a two-tailed independent-samples *t*-test. The values used for sample size estimation were derived from previous research (Xie et al., 2022b). Using G^*^Power, an effect size of Cohen's *d* = 0.843 with α = 0.05 and power = 0.80 indicated that 24 participants were required per group. Allowing for an anticipated dropout rate of approximately 20%, the target sample size was increased to 30 participants per group, yielding a planned total sample size of 60 participants. Because of the characteristics of neuroimaging studies, no unified standard exists for fMRI sample size estimation. However, previous studies (Desmond and Glover, 2002; Hayasaka et al., 2007) suggest that 12–15 participants may be sufficient to detect significant effects. Because this was a pilot neuroimaging trial, the sample size was primarily powered for the clinical fatigue outcome and was not designed to provide definitive power for voxel-wise fMRI outcomes. Therefore, neuroimaging findings should be interpreted as exploratory and require replication in larger samples.

Sixty participants were randomized (30 to the Tai Chi group and 30 to the health education group). During the study, one participant in the health education group withdrew, and two participants were excluded from the final imaging analysis because of excessive head motion (>2.5 mm), one from the health education group and one from the Tai Chi group. A total of 57 participants completed both the fMRI scans and fatigue assessments and were included in the complete-case analysis. No interim analyses were planned or performed, and no formal stopping guidelines were specified because this was a 12-week pilot neuroimaging trial.

### 2.5 Intervention

#### 2.5.1 Tai Chi group

**Figure 1:**
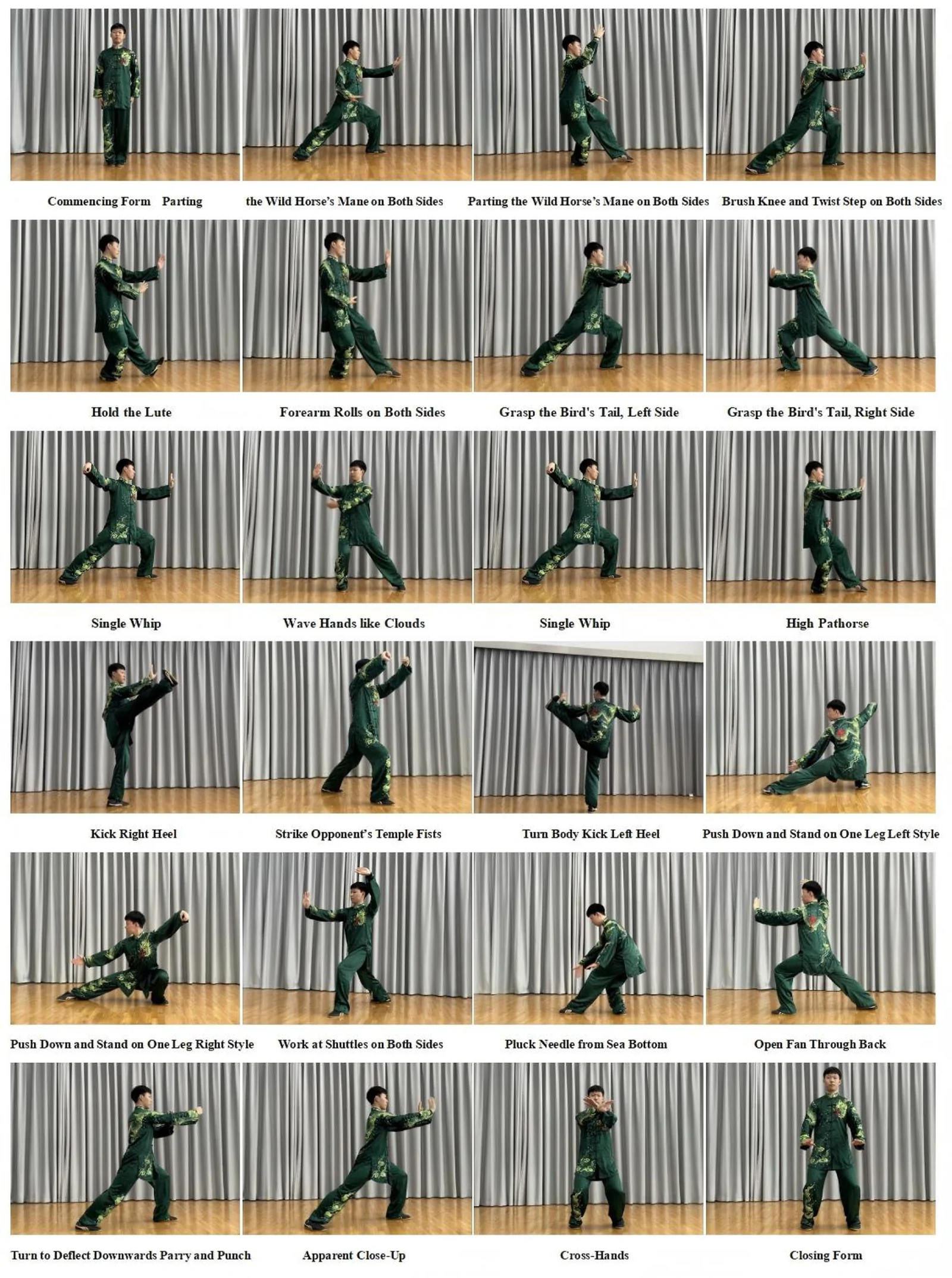
Representative sequence of movements in the standardized 24-form Tai Chi intervention.

Participants in the Tai Chi group were trained in the 24-form Tai Chi by an experienced instructor. The 24-form Tai Chi movements are shown in Figure 1. The intervention lasted 12 weeks and consisted of three sessions per week, including two in-person sessions and one supervised online session. Each session lasted 60 min and included four stages:

Step 1. Warm-up (5 min):

Joint mobility and stretching exercises involving the head, trunk, and limbs.

Step 2. Standing postures (10 min):

Basic postures, including Wuji and related forms. Participants were instructed to remain relaxed, breathe evenly, and focus their attention on the dantian.

Step 3. Forms practice (40 min):

Participants practiced the 24-form sequence, including review of previously learned movements and instruction in new movements. The instructor provided demonstrations and corrective feedback. Participants were allowed to rest if they experienced any discomfort.

Step 4. Cool-down (5 min):

Relaxation exercises included gentle tapping and controlled breathing to gradually return the heart rate to baseline.

During home practice, sessions were supervised through WeChat video calls. Participants completed a practice log after each session. Exercise intensity was maintained at a low-to-moderate level, and participants were instructed to avoid overexertion and to rest immediately if post-exertional malaise, dizziness, pain, or marked discomfort occurred. Session attendance was recorded by the instructor. Intervention adherence was calculated as the number of completed sessions divided by the 36 planned sessions. Participants who completed at least 80% of sessions were considered adherent. Participants were asked not to initiate new structured exercise programs, rehabilitation programs, or fatigue-related treatments during the study period.

#### 2.5.2 Health education group

Participants received CFS-related education through WeChat three times per week. The health education curriculum included weekly topics on CFS symptom recognition, sleep hygiene, pacing and energy conservation, nutrition, stress management, and relapse prevention. Each session lasted approximately 60 min and was delivered using standardized written materials and WeChat-based discussion. Participants were asked to read the materials, complete a brief learning log, and avoid initiating new exercise or rehabilitation programs. The intervention lasted 12 weeks. The health education group controlled for disease-related information exposure and online contact but did not match the Tai Chi group for physical activity, movement training, instructor feedback, or exercise-related expectancy. Accordingly, this comparator was designed to control for disease-related information exposure and study contact rather than to isolate the specific effects of Tai Chi from those of light physical activity, motor learning, instructor attention, or exercise-related contextual effects.

### 2.6 Data acquisition

Imaging data were acquired using a 3.0-T Siemens MRI scanner (Magnetom Viro) with a 32-channel head coil. Resting-state blood oxygen level-dependent (BOLD) signals were acquired using a gradient-echo echo-planar imaging sequence. T1-weighted images were acquired using a magnetization-prepared rapid gradient-echo (MPRAGE) sequence with the following parameters: repetition time (TR) = 7.3 ms, echo time (TE) = 3.1 ms, field of view (FOV) = 230 × 230 mm^2^, flip angle = 10°, matrix = 256 × 256, and slice thickness = 1 mm. Resting-state fMRI data were acquired using an echo-planar imaging sequence with the following parameters: TR = 2,000 ms, TE = 30 ms, FOV = 230 × 230 mm^2^, matrix = 64 × 64, slice thickness = 3.5 mm, 36 slices, and 240 volumes. Participants were instructed to rest with their eyes closed and to keep their heads still during scanning. Participants were instructed not to fall asleep during the scan. Immediately after scanning, participants were asked whether they had fallen asleep. Baseline and post-intervention scans were scheduled at approximately the same time of day for each participant whenever possible to reduce circadian effects. Data were collected before and after the 12-week intervention.

Data preprocessing was performed using MATLAB 2015a, SPM12, and DPABI. Digital Imaging and Communications in Medicine (DICOM) images were converted to Neuroimaging Informatics Technology Initiative (NIfTI) format. The first five volumes were discarded. Head motion correction was applied, and participants with motion exceeding 2.5 mm of translation or 2° of rotation were excluded. Mean framewise displacement (FD) was calculated for each participant. Between-group differences in mean FD were tested at baseline and post-intervention, and mean FD was included as a covariate in group-level imaging analyses. Images were manually reoriented and segmented into gray matter, white matter, and cerebrospinal fluid. Nuisance covariates, including white matter signals, cerebrospinal fluid signals, and head motion parameters (Friston 24), were regressed out. Functional images were normalized to Montreal Neurological Institute (MNI) space with a voxel size of 3 × 3 × 3 mm^3^. Spatial smoothing was performed using a 6-mm Gaussian kernel. The data were detrended and band-pass filtered at 0.01–0.08 Hz.

### 2.7 ALFF calculation

After preprocessing, the time series of each voxel was transformed into the frequency domain using fast Fourier transform. The square root of the power spectrum was averaged across the 0.01–0.08 Hz frequency band to obtain ALFF values. To reduce inter-individual variability, ALFF maps were standardized by dividing each voxel's ALFF value by the global mean ALFF within the brain mask, yielding mALFF maps. The mALFF maps were used in subsequent group-level analyses.

### 2.8 Outcomes

#### 2.8.1 Primary outcome: Multidimensional Fatigue Inventory (MFI-20)

The prespecified primary clinical outcome for this manuscript was fatigue severity measured using the MFI-20 at baseline and immediately after the 12-week intervention. The MFI-20 is a 20-item scale developed by Smets et al. (1995). It assesses general fatigue, physical fatigue, mental fatigue, reduced activity, and reduced motivation. Each dimension includes two items assessing fatigue and two assessing non-fatigue. Reverse scoring was applied to the non-fatigue items according to Smets et al. (1995) to ensure standardization. Items are rated on a 5-point Likert scale (1 = strongly disagree, 5 = strongly agree), yielding a total score of 20–100. Fatigue items are scored positively, whereas non-fatigue items are reverse scored. A higher total score indicates more severe fatigue symptoms (Lundh Hagelin et al., 2007). The MFI-20 has good internal consistency (Cronbach's α = 0.89) and reliability (Pearson correlation coefficient = 0.73) (Chung et al., 2014).

#### 2.8.2 Secondary outcome: amplitude of low-frequency fluctuations (ALFF)

The prespecified secondary neuroimaging outcome for this manuscript was ALFF derived from resting-state fMRI at baseline and immediately after the 12-week intervention. ALFF is a commonly used metric in fMRI studies. It is used to quantify the intensity of low-frequency brain activity, which is closely related to spontaneous neural activity. ALFF measures the amplitude of low-frequency signals and reflects the level of spontaneous activity in each voxel at rest. It reflects the strength of spontaneous neuronal activity in specific brain regions. ALFF analysis can suppress non-specific signals and improve neuronal specificity for detecting spontaneous brain activity (Zhang et al., 2021). As a reliable measure of intrinsic brain activity, ALFF has been widely used in studies of brain function, neurodegenerative diseases, psychiatric disorders, and cognitive neuroscience.

#### 2.8.3 Harms assessment

Harms were defined as any unfavorable or unintended symptom, sign, injury, or medical event occurring during the intervention period, regardless of its presumed relationship to the assigned intervention. Participants were instructed to record unfavorable symptoms experienced after intervention sessions in weekly logs, with particular attention to worsening fatigue or suspected post-exertional malaise, dizziness, pain, falls, marked discomfort, missed sessions, and events requiring medical attention. Instructors also monitored participants for visible discomfort during supervised sessions. A validated PEM-specific questionnaire was not administered. Because PEM may have a delayed onset, the monitoring procedures may not have captured all post-session PEM occurring between supervised sessions or weekly assessments.

### 2.9 Statistical analysis

Data were analyzed using IBM SPSS Statistics version 21.0 (IBM Corp., Armonk, NY, USA). Continuous variables, such as age and scale scores, are presented as mean ± standard deviation (SD). Independent-samples *t*-tests were used for normally distributed data with equal variances, whereas Mann–Whitney *U* tests were used for non-normally distributed data. For non-imaging analyses, a two-sided *P* value < 0.05 was considered statistically significant. To assess differences in brain activity, two-sample *t*-tests were performed on ALFF maps for between-group comparisons, and paired *t*-tests were used for within-group comparisons in SPM12. Statistical significance for imaging analyses was defined using family-wise error (FWE) correction. Pearson correlation analyses were performed to examine the associations between changes in ALFF within significant brain regions and changes in MFI-20 scores. Effect sizes (Cohen's *d* for between-group comparisons) were calculated to estimate the magnitude of the treatment effect. Imaging results were corrected for multiple comparisons using cluster-level family-wise error (FWE) correction. A voxel-level threshold of *P* < 0.001 was first applied as the cluster-forming threshold, and statistical significance was then defined as cluster-level FWE-corrected *P* < 0.05. Age, sex, and years of education were included as covariates. The primary analysis population for this manuscript comprised randomized participants with available baseline and post-intervention MFI-20 data and usable fMRI data. Participants were analyzed according to the group to which they were randomized. Missing post-intervention data and unusable fMRI scans were handled by complete-case analysis; no imputation was performed for the analyses reported in this manuscript. Correlation analyses between ALFF changes and MFI-20 changes were exploratory and were interpreted as ancillary brain-behavior analyses.

## 3 Results

### 3.1 Baseline comparison between the two groups

Before randomization, 66 patients were assessed for eligibility, six were excluded before randomization because they did not meet eligibility criteria (*n* = 4), declined participation (*n* = 2). Sixty participants were randomized, with 30 allocated to Tai Chi and 30 allocated to health education. One participant in the health education group withdrew after randomization, and two participants were excluded from the final imaging analysis because of excessive head motion (>2.5 mm), one from each group. Therefore, 57 participants were included in the final complete-case analyses (Tai Chi group, *n* = 29; health education group, *n* = 28). Recruitment began in April 2024, and post-intervention outcome assessment was completed by January 2025. The trial ended after the planned 12-week intervention and outcome assessments were completed; it was not stopped early.

**Figure 2:**
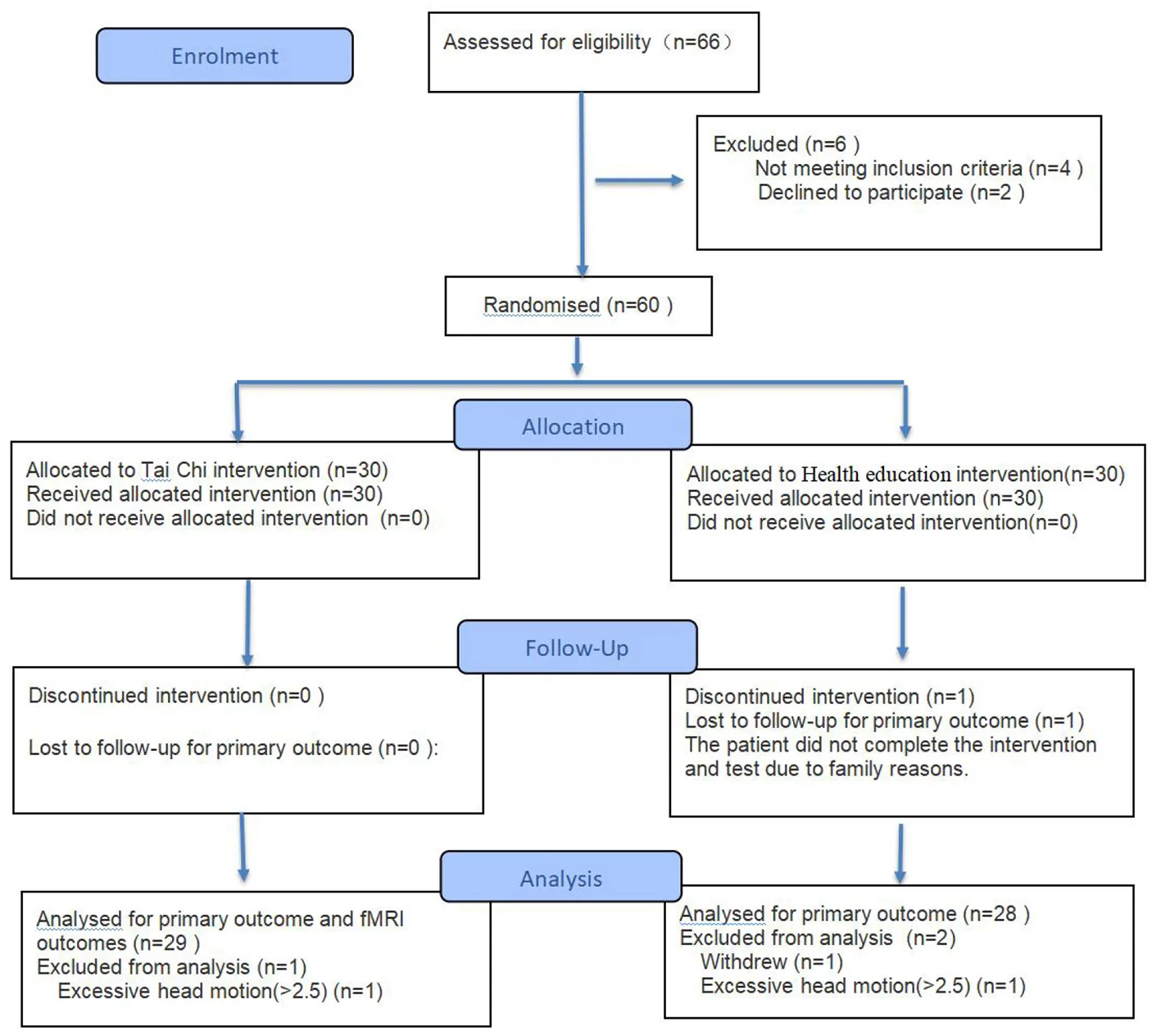
Flow diagram of participant recruitment, randomization, intervention, and analysis.

The Tai Chi group comprised six men and 23 women, with a mean age of 39.17 ± 9.75 years, mean height of 163.48 ± 9.09 cm, mean weight of 64.21 ± 13.06 kg, mean body mass index (BMI) of 23.85 ± 3.23, mean education duration of 17.48 ± 2.28 years, mean MFI-20 of 52.28 ± 5.45, and mean PSQI of 7.76 ± 1.96. The health education group comprised nine men and 19 women, with a mean age of 35.89 ± 9.21 years, mean height of 166.29 ± 6.73 cm, mean weight of 65.96 ± 13.38 kg, mean BMI of 23.68 ± 3.56, mean education duration of 17.71 ± 2.18 years, mean MFI-20 of 52.96 ± 4.17, and mean PSQI of 7.61 ± 1.73. Baseline demographic and clinical characteristics, along with between-group comparisons, are shown in Table 1. The study flow diagram is shown in Figure 2. No significant between-group differences were observed in sex, age, height, weight, BMI, years of education, MFI-20 or PSQI (*P* > 0.05), indicating comparable baseline characteristics.

**Table 1:** Baseline demographic and clinical characteristics of the complete-case analysis population.

Variable	Tai Chi group (*n* = 29)	Health education group (*n* = 28)	*P*-value
Sex, male/female	6/23	9/19	0.50
Age, years	39.17 ± 9.75	35.89 ± 9.21	0.20
Height, cm	163.48 ± 9.09	166.29 ± 6.73	0.19
Weight, kg	64.21 ± 13.06	65.96 ± 13.38	0.62
BMI	23.85 ± 3.23	23.68 ± 3.56	0.85
Years of education	17.48 ± 2.28	17.71 ± 2.18	0.70
MFI-20	52.28 ± 5.45	52.96 ± 4.17	0.59
PSQI	7.76 ± 1.96	7.61 ± 1.73	0.76

Data are presented as mean ± SD, except for sex, which is presented as n. The table includes participants with available baseline and postintervention MFI-20 data and usable paired fMRI data.

### 3.2 MFI-20 scores

**Figure 3:**
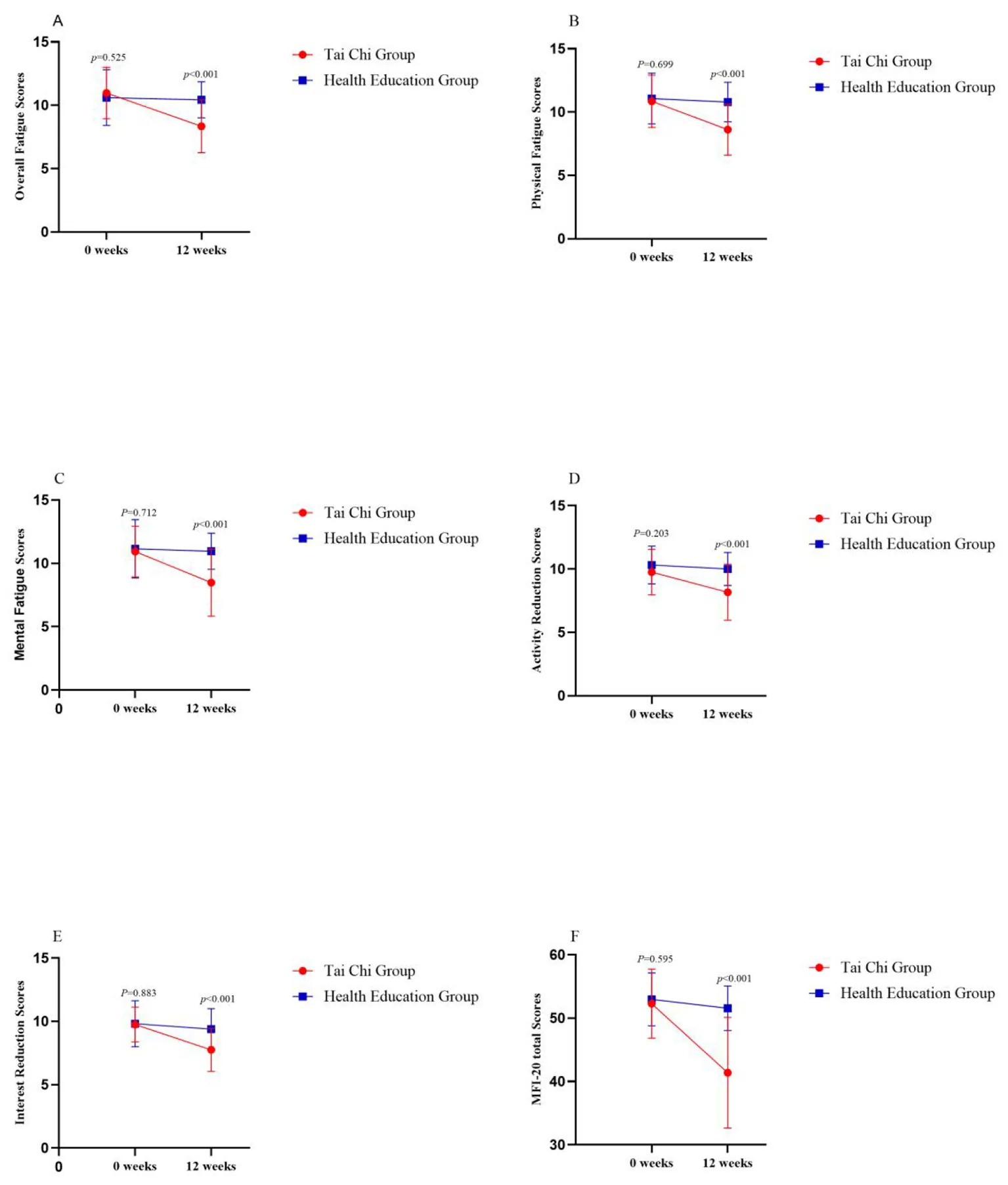
Effects of Tai Chi on fatigue at baseline and week 12. Panels **A–F** show the MFI-20 dimension scores, including overall fatigue **(A)**, physical fatigue **(B)**, mental fatigue **(C)**, activity reduction **(D)**, interest reduction **(E)**, and total score **(F)**, for the Tai Chi group and Health Education group at baseline (0 weeks) and week 12.Data are presented as mean ± standard deviation (SD). *P* values indicate between-group differences at each time point.

After 12 weeks, the Tai Chi group showed significant reductions in overall fatigue, physical fatigue, mental fatigue, activity reduction, and interest reduction (*P* < 0.001; Figure 3), whereas no significant changes were observed in the health education group (*P* > 0.05). Between-group comparisons of change scores revealed significantly greater improvements in the Tai Chi group across all MFI-20 dimensions. Overall fatigue: −2.44 (−3.64, −1.25), Cohen's *d* = −1.12, *P* < 0.001; physical fatigue: −1.96 (−3.21, −0.71), Cohen's *d* = −0.85, *P* = 0.002; mental fatigue: −2.27 (−3.51, −1.03), Cohen's *d* = −1.00, *P* < 0.001;Activity reduction: −1.26 (−2.18, −0.35), Cohen's *d* = −0.75, *P* = 0.007; interest reduction: −1.57 (−2.74, −0.40), Cohen's *d* = −0.74, *P* = 0.007; MFI-20 total score: −9.50 (−12.31, −6.70), Cohen's *d* = −1.83, *P* < 0.001 (Table 2). Lower scores indicate less severe fatigue. These findings indicate that Tai Chi produces substantial and statistically significant improvements in fatigue relative to health education.

**Table 2:** Comparison of MFI-20 scores before and after the intervention in both groups.

Dimension	Tai Chi baseline Mean ±SD	Tai Chi week 12 Mean ±SD	Health Ed baseline Mean ±SD	Health Ed week 12 Mean ±SD	Between-group difference (95% CI)	Cohen's d	*P* value
Overall fatigue	10.97 ± 2.03	8.34 ± 2.09	10.61 ± 2.20	10.43 ± 1.43	−2.44 (−3.64, −1.25)	−1.12	< 0.001
Physical fatigue	10.86 ± 2.07	8.62 ± 2.01	11.07 ± 2.00	10.79 ± 1.57	−1.96 (−3.21, −0.71)	−0.85	0.002
Mental fatigue	10.93 ± 2.00	8.48 ± 2.65	11.14 ± 2.30	10.96 ± 1.43	−2.27 (−3.51, −1.03)	−1.00	< 0.001
Activity reduction	9.76 ± 1.79	8.17 ± 2.21	10.32 ± 1.49	10.00 ± 1.31	−1.26 (−2.18, −0.35)	−0.75	0.007
Interest reduction	9.76 ± 1.38	7.76 ± 1.70	9.82 ± 1.81	9.39 ± 1.62	−1.57 (−2.74, −0.40)	−0.74	0.007
MFI-20 total scores	52.28 ± 5.45	41.38 ± 8.74	52.96 ± 4.17	51.57 ± 3.54	−9.50 (−12.31, −6.70)	−1.83	< 0.001

Data are presented as mean ± standard deviation (SD). Between-group differences are expressed as mean differences with 95% confidence intervals (95% CI). Cohen's d was calculated to assess effect size. P values represent between-group comparisons of the changes from baseline.

### 3.3 Changes in ALFF

**Figure 4:**
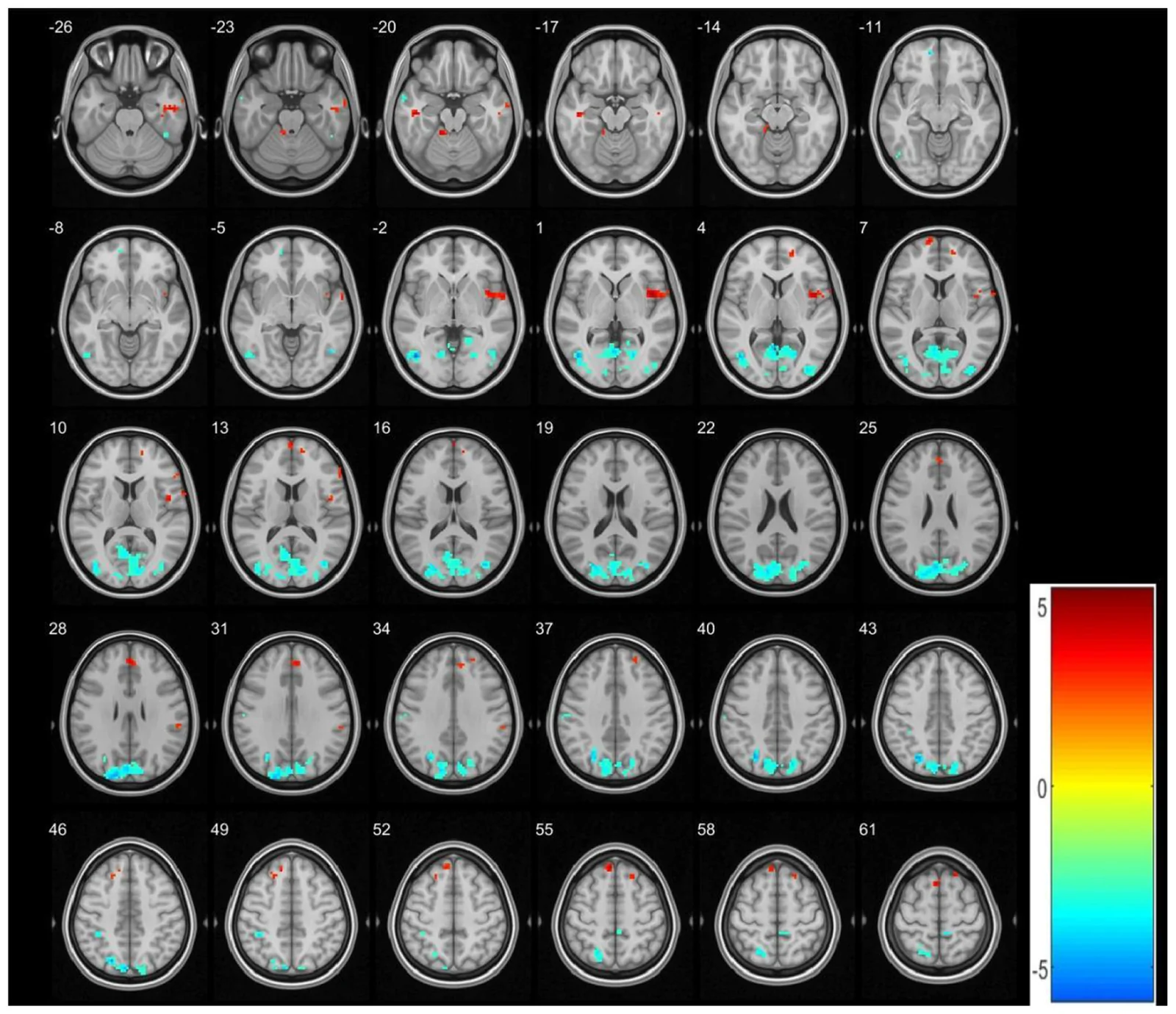
Brain regions showing significant ALFF changes after a 12-week Tai Chi intervention in patients with CFS. Warm colors indicate increased ALFF, and cool colors indicate decreased ALFF. Statistical thresholds were set at the voxel level at *P* < 0.001 and at the cluster level at *P* < 0.05, with family-wise error correction.

Compared with baseline, the Tai Chi group showed increased ALFF after 12 weeks in the bilateral medial superior frontal gyri, right dorsolateral superior frontal gyri, and left supplementary motor area (voxel-level *P* < 0.001 with cluster-level FWE correction at *P* < 0.05). ALFF decreased in the left inferior occipital gyrus, right inferior temporal gyrus, left supramarginal gyrus, and left superior parietal gyrus (voxel-level *P* < 0.001 with cluster-level FWE correction at *P* < 0.05). These results are presented in Table 3 and Figure 4.

**Table 3:** Brain regions showing ALFF differences after 12 weeks of Tai Chi intervention in patients with CFS.

Brain region	Hemisphere	Cluster size/K	MNI coordinates			Peak *t* value
			X	Y	Z	
SMA	L	57	−3	15	66	5.4235
SFGmed	L	24	0	45	27	3.8722
SFGmed	R	14	12	57	15	3.3827
SFGdor	R	13	15	0	72	3.8568
IOG	L	1,212	−45	−69	−3	−5.8745
SPG	L	38	−21	−63	54	−3.8709
SMG	L	14	−39	−42	48	−4.1612
ITG	R	10	45	−63	−6	−4.2942

Coordinates are reported in MNI space. Positive T values indicate increased ALFF, and negative T values indicate decreased ALFF.

ALFF, amplitude of low-frequency fluctuations; CFS, chronic fatigue syndrome; IOG, inferior occipital gyrus; ITG, inferior temporal gyrus; L, left; R, right; SFGdor, dorsolateral superior frontal gyrus; SFGmed, medial superior frontal gyrus; SMA, supplementary motor area; SMG, supramarginal gyrus; SPG, superior parietal gyrus.

**Figure 5:**
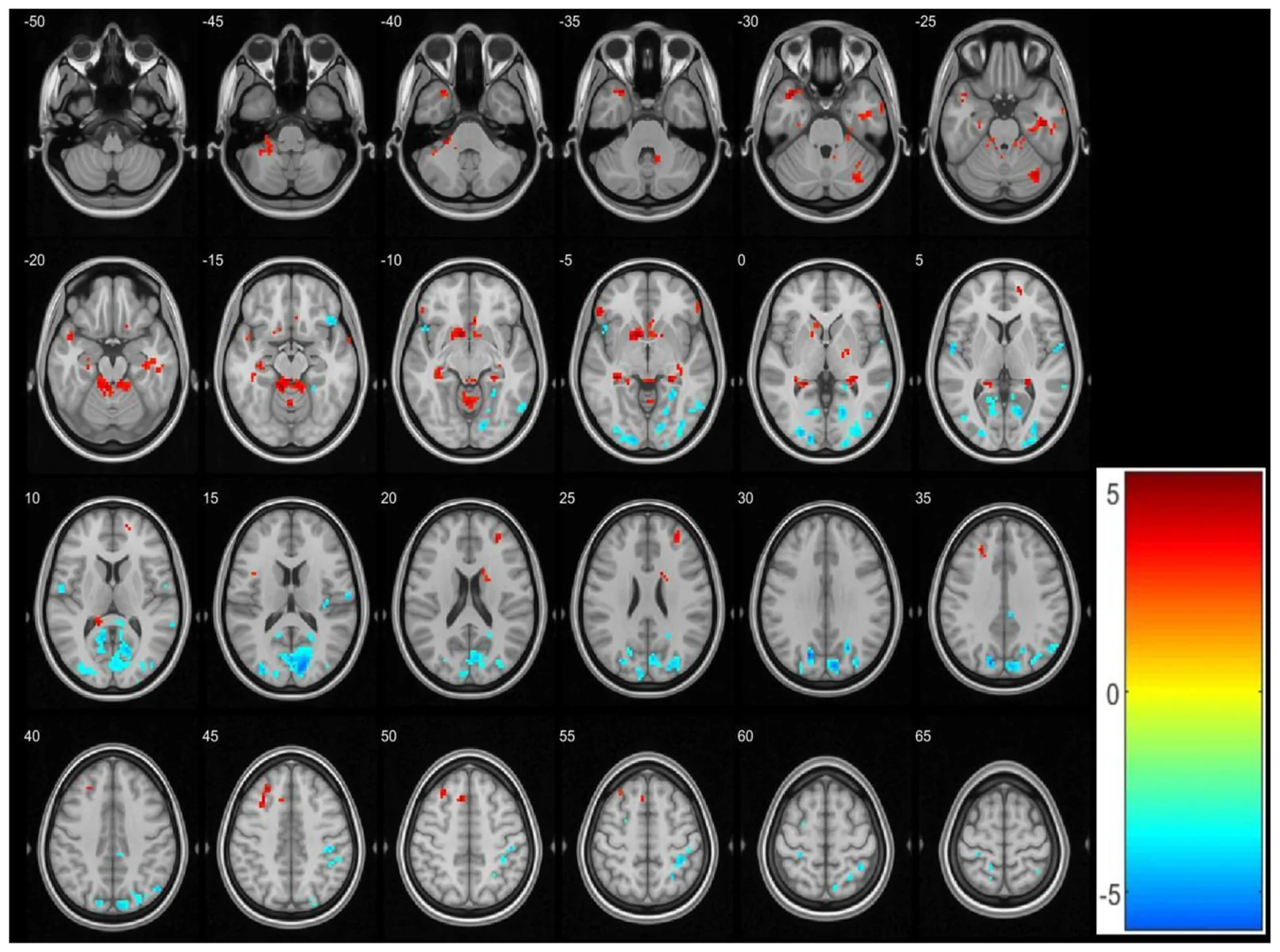
Brain regions showing significant ALFF differences relative to the health education intervention in patients with CFS. Warm colors indicate increased ALFF, and cool colors indicate decreased ALFF. Statistical thresholds were set at the voxel level at *P* < 0.001 and at the cluster level at *P* < 0.05, with family-wise error correction.

Compared with the health education group, after the intervention, the Tai Chi group showed increased ALFF in the left and right hippocampi, left caudate nucleus, bilateral middle frontal gyri, and left supplementary motor area (voxel-level *P* < 0.001 with cluster-level FWE correction at *P* < 0.05). In contrast, the Tai Chi group showed decreased ALFF in the left and right superior occipital gyri, right middle temporal gyrus, left superior temporal gyrus, and left middle occipital gyrus (voxel-level *P* < 0.001 with cluster-level FWE correction at *P* < 0.05). These between-group differences are presented in Table 4 and Figure 5.

**Table 4:** Brain regions showing between-group ALFF differences after 12 weeks of intervention in patients with CFS.

Brain region	Hemisphere	Cluster size/K	MNI coordinates			Peak *t* value
			X	Y	Z	
Hipp	L	87	−33	−33	−6	4.2448
Hipp	R	33	27	−36	3	3.8796
Caud	L	48	−9	12	−9	4.6386
MFG	L	47	−27	33	45	3.6669
OFMidG	R	14	6	24	−9	3.8748
SMA	L	13	9	24	51	3.6882
SOG	R	117	24	−66	30	−4.3521
SOG	L	49	−18	−72	30	−4.9217
MTG	R	31	45	−69	0	−3.4722
MOG	L	21	−27	−78	24	−3.626
STG	L	17	−54	−3	6	−4.1781

Coordinates are reported in MNI space. Positive T values indicate higher ALFF in the Tai Chi group than in the health education group, whereas negative T values indicate lower ALFF in the Tai Chi group than in the health education group. ALFF, amplitude of low-frequency fluctuations; CFS, chronic fatigue syndrome; Caud, caudate nucleus; Hipp, hippocampus; L, left; MFG, middle frontal gyrus; MOG, middle occipital gyrus; MTG, middle temporal gyrus; OFMidG, orbital part of the middle frontal gyrus; R, right; SMA, supplementary motor area; SOG, superior occipital gyrus; STG, superior temporal gyrus.

### 3.4 Correlation between ALFF and MFI-20

**Figure 6:**
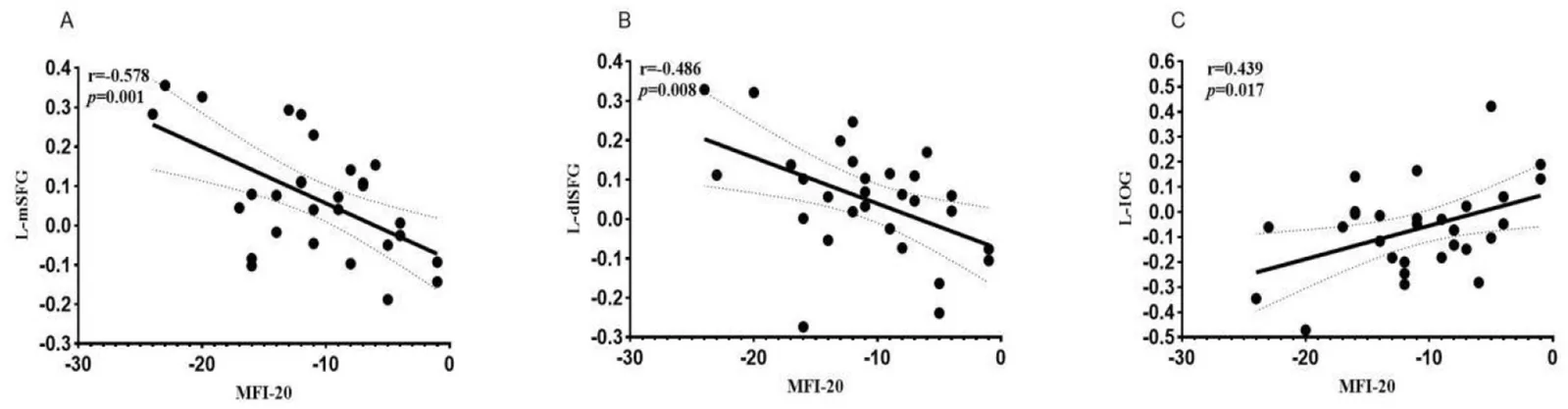
Exploratory associations between changes in mean-normalized amplitude of low-frequency fluctuations and changes in MFI-20 total scores in the Tai Chi group. **(A)** Left medial superior frontal gyrus (L-mSFG); **(B)** left dorsolateral superior frontal gyrus (L-dlSFG); and **(C)** left inferior occipital gyrus (L-IOG). Change scores were calculated as week 12 minus baseline; therefore, negative ΔMFI-20 values indicate reductions in fatigue severity. Solid lines represent least-squares regression lines, and dashed lines represent 95% confidence intervals. Pearson correlation coefficients and *P* values are displayed in each panel. ALFF, amplitude of low-frequency fluctuations; MFI-20, Multidimensional Fatigue Inventory.

Changes in ALFF in the left medial superior frontal gyrus (L-mSFG) and left dorsolateral superior frontal gyrus (L-dlSFG) were negatively correlated with changes in MFI-20 scores (*r* = −0.578 and *r* = −0.486, respectively; *P* < 0.05). Changes in ALFF in the left inferior occipital gyrus (L-IOG) were positively correlated with changes in MFI-20 scores (*r* = 0.439, *P* < 0.05). The results of the correlation analyses are shown in Figure 6.

### 3.5 Adverse events

No adverse events were reported through the weekly participant logs or observed during supervised sessions. Specifically, no falls, injuries, dizziness requiring intervention discontinuation, or severe worsening described by participants as post-exertional malaise was reported through these monitoring procedures. However, because a validated PEM-specific instrument and scheduled delayed post-session assessments were not used, these findings indicate an absence of reported severe events under the study monitoring procedures rather than definitive evidence that no PEM occurred. Within these limitations, the intervention was generally well-tolerated by the participants who completed the study.

## 4 Discussion

In this randomized parallel-group neuroimaging study, a 12-week Tai Chi intervention was associated with greater improvements in multidimensional fatigue symptoms than health education. At the neural level, Tai Chi was associated with altered ALFF in prefrontal, motor, hippocampal/striatal, and sensory-related regions. Exploratory brain–behavior analyses suggested that changes in prefrontal and occipital ALFF were associated with fatigue improvement. These findings provide preliminary evidence that Tai Chi-related fatigue improvement in CFS may be accompanied by region-specific changes in spontaneous brain activity.

In the present study, a 12-week Tai Chi intervention was associated with reduced fatigue symptoms in patients with CFS. MFI-20 scores decreased across all dimensions, with greater improvements than those observed in the health education group. These findings are consistent with previous evidence suggesting that Tai Chi and other mind–body interventions may alleviate fatigue (Guo et al., 2014). Compared with health education, Tai Chi was associated with both clinical improvement and measurable changes in brain activity, suggesting that its effects may involve central nervous system regulation.

However, because the health education comparator did not match the Tai Chi intervention for physical movement, motor-skill acquisition, instructor feedback, exercise-related social interaction, or treatment expectancy, the present trial cannot determine whether the observed fatigue and ALFF changes were attributable specifically to the meditative or mind–body components of Tai Chi. The findings may also reflect non-specific effects of low-intensity physical activity, repeated motor practice, instructor attention, expectancy, or a combination of these factors. A future three-arm trial comparing Tai Chi with both an intensity- and contact-matched light-exercise intervention and a health education intervention would be needed to distinguish Tai Chi-specific mind–body effects from general exercise and contextual effects.

The present findings should be interpreted as complementary to previous regional and network-level neuroimaging studies rather than as the first demonstration of brain functional changes following Tai Chi in CFS. A 12-week PLWNT Qigong study reported changes in ALFF and seed-based functional connectivity, whereas previous 1-month Tai Chi studies demonstrated altered effective connectivity between resting-state networks and changes in whole-brain functional connectivity involving the default mode and frontoparietal networks. Unlike those Tai Chi studies, the present trial used a randomized health education-controlled design, extended the intervention to 12 weeks, applied a standardized 24-form protocol, and examined whether regional mALFF changes were associated with multidimensional fatigue improvement. Thus, the principal contribution of this study is the characterization of local spontaneous brain activity under randomized parallel-group conditions, rather than the initial identification of large-scale network effects.

From a neuroimaging perspective, Tai Chi was associated with region-specific alterations in spontaneous brain activity. Increased ALFF was observed in the bilateral medial superior frontal gyri, bilateral dorsolateral superior frontal gyri, and left supplementary motor area, whereas decreased ALFF was observed in occipital, temporal, and parietal regions. These neural changes were associated with improvements in fatigue symptoms, suggesting a potential neurobiological basis for the observed effects of Tai Chi.

The observed increases in ALFF within the medial and dorsolateral superior frontal gyri may reflect enhanced central executive network (CEN) function, which supports cognitive control, attention regulation, and goal-directed behavior (Cook et al., 2007; Mizuno et al., 2015; Van Der Schaaf et al., 2017; Sandoval et al., 2025). In addition, reduced gray matter volume in the prefrontal cortex has been reported in CFS and has been shown to correlate with fatigue severity (Barnden et al., 2015), whereas cognitive behavioral therapy has been associated with increased prefrontal volume and improved cognitive performance (De Lange et al., 2008). These findings suggest that Tai Chi may influence prefrontal regions associated with the central executive network (CEN), potentially enhancing top-down regulation of fatigue-related cognitive and affective processes. In contrast, decreased ALFF in sensory and visual regions may reflect reduced inefficient compensatory neural activity and a shift toward a more balanced neural state. This pattern may contribute to symptom relief in patients with CFS.

The hippocampus, a critical component of the limbic system, is highly sensitive to stress, and prolonged exposure to glucocorticoids can induce structural and functional alterations that affect emotional processing and cognition (Kim et al., 2015). Bidirectional circuitry between the hippocampus and the medial prefrontal cortex (mPFC) supports emotion regulation, episodic memory, and cognitive control. In our study, increased hippocampal ALFF may be related to altered limbic activity. Because functional connectivity was not directly tested, the interpretation regarding hippocampus–mPFC network function remains speculative, consistent with prior findings showing strengthened resting-state connectivity and improved memory performance following Tai Chi intervention (Tao et al., 2016; Jin and Maren, 2015). These findings provide convergent support for the present observation of increased hippocampal ALFF, which may reflect strengthened hippocampus-related network function.

The observed increase in ALFF within the caudate nucleus is also noteworthy. As a major component of the dorsal striatum, the caudate nucleus is closely associated with reward processing, motivation, and goal-directed behavior (Nakamura and Hikosaka, 2006). Therefore, enhanced spontaneous activity in this region may reflect upregulation or restoration of reward–motivation circuitry. Previous studies in CFS have reported reduced basal ganglia or striatal responsiveness, which is closely linked to fatigue symptoms (Miller et al., 2014). This finding suggests that fatigue is not merely a physical phenomenon but also involves dysfunction in motivational and action-related neural systems. By promoting motor fluency and controlled movement, Tai Chi practice may repeatedly engage these circuits, thereby enhancing motivation and contributing to fatigue reduction.

We also observed increased ALFF in the left supplementary motor area (SMA), a region involved in motor planning and voluntary movement. Tai Chi emphasizes coordinated, continuous movements and balance control, which require integration of motor planning and execution. The observed increase in SMA activity may reflect training-related neuroplasticity within motor networks associated with repeated practice and may contribute to improved movement efficiency and reduced fatigue burden.

In contrast, decreased ALFF was observed in occipital, temporal, and parietal regions, including the inferior and superior occipital gyri, middle occipital gyrus, inferior and superior temporal gyri, middle temporal gyrus, and supramarginal gyrus. These regions are primarily involved in visual processing, sensory integration, and attentional allocation. Importantly, decreased ALFF does not necessarily indicate impaired function; rather, it may reflect reduced abnormal hyperactivity, improved neural efficiency, or a shift toward a more stable functional state.

Previous studies have reported functional abnormalities in occipital regions in CFS (Leong et al., 2022; Ohinata et al., 2008). A study of the “Yannian Jiuzhuan Method” reported reduced ALFF in the left occipital and temporal regions after intervention (Xie et al., 2022b), consistent with our findings. Tai Chi emphasizes internal attention and body awareness, which may reduce reliance on external visual input and thereby decrease spontaneous activity in visual regions.

Similarly, decreased ALFF in the right inferior temporal gyrus may reflect reduced cognitive load related to visual semantic processing and object recognition. The temporal lobe is involved in emotion, sleep, and cognition, and its dysfunction has been linked to fatigue-related symptoms (Kuratsune et al., 2002). A previous study reported reduced fractional ALFF (fALFF) in the temporal lobe following Tai Chi intervention in patients with CFS (Li et al., 2023), further supporting our findings.

In addition, decreased ALFF in the left supramarginal gyrus and parietal regions may indicate improved efficiency of sensory integration and attentional processing. Given that patients with CFS often exhibit deficits in attention and multisensory integration, normalization of activity in these regions may contribute to symptom improvement.

The correlation analyses further support an association between brain activity and fatigue symptoms. Increased ALFF in prefrontal regions was negatively correlated with fatigue severity, suggesting that enhanced executive control may contribute to symptom relief. In contrast, decreased ALFF in the occipital cortex was positively correlated with fatigue reduction, suggesting that reduced sensory-related activity may be associated with improved neural efficiency. Together, these findings suggest that Tai Chi may be associated with functional reorganization across brain networks, characterized by enhanced prefrontal regulation and reduced aberrant activity in sensory-related regions.

From a broader perspective, these results are consistent with the concept of neuroplasticity, whereby repeated engagement in mind–body practices may reshape intrinsic brain activity. Previous randomized controlled studies have reported that Tai Chi and Baduanjin may increase low-frequency fluctuations in prefrontal regions and improve cognitive function in older adults (Tao et al., 2017). Our findings extend this evidence to patients with CFS and suggest that similar neural mechanisms may underlie symptom improvement across different populations.

From a clinical standpoint, Tai Chi is a low-cost, accessible, and well-tolerated intervention that may be suitable for patients with CFS, who often cannot tolerate high-intensity exercise. Mind–body interventions such as Tai Chi and Qigong integrate movement, breathing, and mental focus and have been shown to improve both physical and psychological wellbeing (Roswiyani et al., 2020). The present neuroimaging findings provide preliminary objective evidence supporting their clinical utility.

Several limitations should be acknowledged. First, the diagnosis of CFS relied partly on self-reported symptoms, introducing potential variability. Second, participant blinding was not feasible, and the health education comparator did not match the Tai Chi intervention for physical activity, instructor feedback, motor-skill learning, or exercise-related expectancy. Therefore, the observed between-group differences cannot be attributed specifically to the mind–body components of Tai Chi and may partly reflect non-specific exercise, attention, or expectancy effects. Third, because the primary clinical outcome, the MFI-20, was entirely self-reported, participants' awareness of treatment allocation could have influenced symptom reporting and may have inflated the apparent between-group benefit. Although the fMRI analyses provide a concurrently measured biological outcome and were performed using coded identifiers, the imaging findings do not eliminate expectation bias in the self-reported outcome and should not be interpreted as proof that the neural changes mediated fatigue improvement. Fourth, the absence of a healthy control group limits interpretation of the observed ALFF changes as normalization vs. compensatory processes. Fifth, the analyses were based on complete cases with usable paired fMRI data; therefore, attrition and imaging-quality exclusions may have introduced selection bias. Sixth, the present manuscript reports the 12-week fatigue and ALFF outcomes, whereas other protocol-listed cognitive and follow-up outcomes are not reported here, the present manuscript should therefore be interpreted as a focused ALFF report rather than the complete trial report. Seventh, ALFF captures local spontaneous activity but does not characterize network-level interactions, and the brain-behavior correlations were exploratory and should be interpreted cautiously. Eighth, PEM was not assessed using a validated PEM-specific instrument or scheduled assessments during the hours and days following each intervention session. Because PEM may have delayed onset, the weekly logs and supervised-session monitoring may have underdetected delayed or transient post-exertional symptom exacerbation. Future studies should replicate and extend the existing network-level literature by using adequately powered samples, prespecified connectivity analyses of networks such as the default mode, frontoparietal, sensorimotor, and salience networks, and active exercise comparators matched for intensity, instructor contact, and expectancy. Formal longitudinal mediation analyses are also needed to determine whether regional or network-level brain changes statistically mediate fatigue improvement.

## 5 Conclusion

In conclusion, this study showed that Tai Chi was associated with reduced fatigue symptoms and altered spontaneous brain activity in patients with CFS. These effects may be associated with increased spontaneous activity in prefrontal regions and decreased activity in sensory-related regions, potentially reflecting functional adaptations related to fatigue improvement. These findings provide preliminary neuroimaging evidence supporting Tai Chi as a potential non-pharmacological intervention for CFS.

## Data Availability

The raw data supporting the conclusions of this article will be made available by the authors, without undue reservation.
